# Narrative Review of Classification Systems Describing Laryngeal Vascularity Using Advanced Endoscopic Imaging

**DOI:** 10.3390/jcm12010010

**Published:** 2022-12-20

**Authors:** Peter Kántor, Lucia Staníková, Anna Švejdová, Karol Zeleník, Pavel Komínek

**Affiliations:** 1 Department of Otorhinolaryngology and Head and Neck Surgery, University Hospital Ostrava, 708 52 Ostrava, Czech Republic; 2 Department of Craniofacial Surgery, Faculty of Medicine, University of Ostrava, 701 03 Ostrava, Czech Republic; 3 Department of Otorhinolaryngology and Head and Neck Surgery, University Hospital Hradec Králove, Faculty of Medicine in Hradec Králove, Charles University, 500 03 Hradec Králové, Czech Republic

**Keywords:** enhanced contact endoscopy, narrow-band imaging, Storz Professional Image Enhancement System, leukoplakia, larynx, laryngeal cancer

## Abstract

Endoscopic methods are critical in the early diagnosis of mucosal lesions of the head and neck. In recent years, new examination methods and classification systems have been developed and introduced into clinical practice. All of these new techniques target the notion of optical biopsy, which tries to assess the nature of the lesion before histology examination. Many methods suffer from interpretation issues due to subjective interpretation of the findings. Therefore, multiple classification systems have been developed to assist the proper interpretation of mucosal findings and reduce the error rate. They provide various perspectives on the assessment and interpretation of mucosa changes. This article provides a comprehensive and critical view of the available classification systems as well as their advantages and disadvantages.

## 1. Introduction

Endoscopy of the upper aerodigestive tract has become a common practice in otolaryngology and remains an inseparable part of in-office diagnostics of head and neck cancer. Nowadays, laryngeal squamous cell carcinoma is the most common form of head and neck cancer [1]. Unfortunately, the mucosal changes caused by a malignant tumor in the early stages are usually small and similar to non-neoplastic lesions. Therefore, differentiating between neoplastic and non-neoplastic tissue changes remains a diagnostic challenge even for experienced clinicians. Moreover, every surgical intervention in the larynx may lead to the deterioration of the voice after surgery due to the scarring of the vocal cords [2]. If a malignant tumor is present, then a resection margin of the healthy tissue is often required to successfully remove the lesion [3]. Therefore, advanced endoscopy methods are needed to identify patients who can be treated with less aggressive surgery or who can even be managed without surgical intervention. Attempting to differentiate between malignant and benign changes with naked eye or regular white light endoscopy is very difficult and histology examination remains the gold standard for the identification of cancerous changes [4]. Thus, many new endoscopy techniques have been developed. These techniques strive towards the concept of pre-histology diagnosis, which tries to determine the lesion histology before the biopsy.

Most methods try to utilize metabolic or morphological tissue changes induced by the lesion. The most popular methods are Narrow Band Imaging^®^ (NBI, Olympus, Tokyo, Japan) or IMAGE 1S^®^ (Karl Storz, Tuttingen, Germany). These methods utilize morphological changes of mucosa vascularization. Changes are caused by the capability of malignant tumors to induce neoangiogenesis. When the tumor is very small, nutrients are supplied to it by simple diffusion from the surrounding extracellular fluid [5]. If the tumor continues to grow, then diffusion becomes insufficient in providing enough nutrients for further cell growth; the tumor thus begins to experience ischemia [5]. Tissue ischemia triggers neoangiogenesis growth factors such as vascular endothelial growth factor (VEGF) [5]. When VEGF comes into contact with endothelial cells, it triggers a signaling cascade initiating the process of neoangiogenesis [5]. The result of this process is the formation of pathological vascularization [5].

Advanced endoscopy imaging methods enhance mucosa vascularization. According to these changes, we can determine with a certain probability if the observed lesion is benign or malignant. A meta-analysis performed by Zhui et al. pooled 25 studies and reported a sensitivity of 88.5% and a specificity 95.6% [6]. Unfortunately, interpretations of the results of the examinations are subjective and therefore may be prone to interpretation errors. One of the possibilities to achieve relative objectivity is to use a classification system. Multiple classification systems have been developed and can be used to determine the character of the laryngeal lesions. They provide interpretation guidelines, which are very useful for the proper assessment of the lesion character. Unfortunately, these classification systems are not uniform, and each has advantages and drawbacks. Thus, the aim of the paper is to provide a complex and critical overview of available classification systems for mucosal laryngeal lesions.

## 2. Materials and Methods

**Figure 1:**
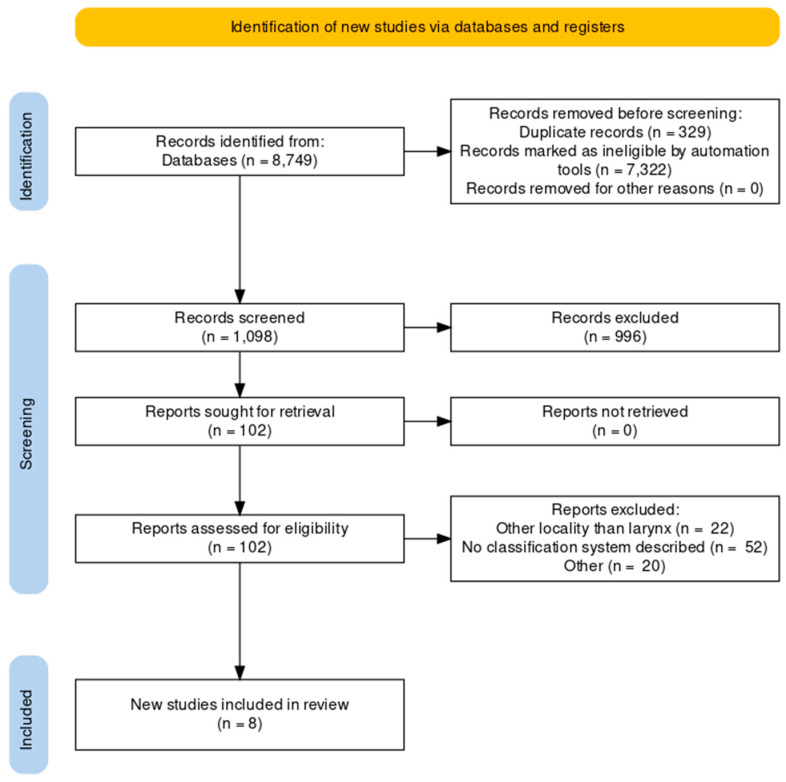
PRISMA flow diagram of the selection process of the relevant articles.

PubMed, the Cochrane Library, and Google Scholar databases were searched using the term “endoscopy”, “head and neck cancer”, “larynx”, and “classification” to identify articles published on the topic within the period 2000–2022. The search was conducted by two independent authors during November 2022. All articles were reviewed and only those written in the English language, dealing with adult patients, and describing a classification system of laryngeal lesions were retained for analysis. All duplicates were removed. Identification of the relevant studies was conducted according to the PRISMA guidelines. The selection process of relevant articles can be seen on the Preferred Reporting Items for Systematic Reviews and Meta-Analyses (PRISMA) flow diagram (Figure 1) [7].

## 3. Results

### 3.1. Current Classification Systems Used in the Description of Laryngeal Mucosal Vascularization

- **Classification according to Ni et al. (2011) [2]**

The first available classification system was described by Ni et al. This classification is widely used by many ENT clinicians and was originally designed to be used with the NBI technology. This classification can be used with other technologies such as IMAGE 1S (Karl Storz) with similar results [3].

**Figure 2:**
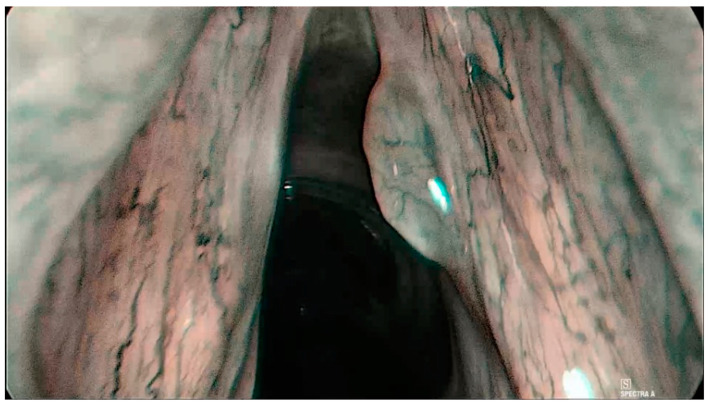
Histologically verified polyp of the right vocal cord, Ni type II of the mucosal vascularization, ELS classification—longitudinal type of vascularization.

**Figure 3:**
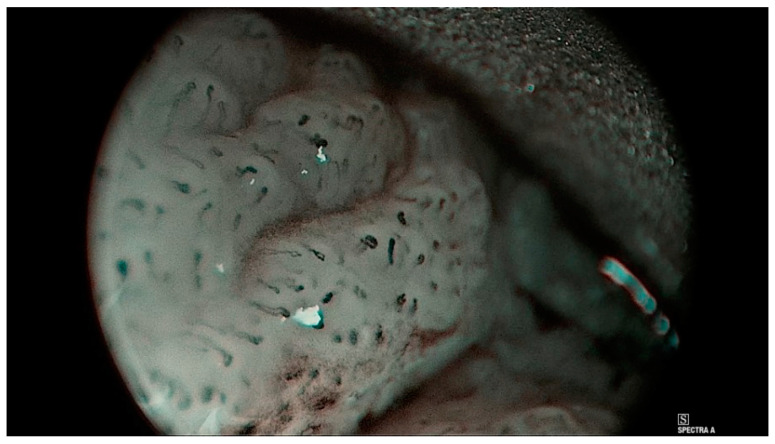
Histologically verified supraglottic squamous cell carcinoma. Ni type Vb of the mucosal vascularization, ELS classification—perpendicular type of vascularization.

This system classifies endoscopy findings according to the changes of intrapapillary capillary loops (IPCLs) into five categories [2]. Lesions in category I–IV are considered to be benign (Figure 2) [2]. Category V lesions are considered malignant lesions and are divided into three subcategories: Va, Vb, and Vc (Figure 3) [2]. Ni et al. reported a cancer lesion detection sensitivity of 88.9% and a specificity of 93.2% [2]. Many subsequent studies and meta-analyses have confirmed the diagnostic value of this classification system [4,5,6,8]. The overview of this classification can be seen in Table 1.

- **Classification proposed by the European Laryngological Society (2016) [9]**

This classification system was published by Arens et al. in 2016 [9]. It separates lesions according to their vascular architecture into two categories: longitudinal or perpendicular [9]. Longitudinal vascularization passes parallel to the mucosa and is associated with benign lesions (Figure 2) [9]. Perpendicular vascularization runs upright in the mucosa and is interpreted as suspicious (Figure 3) [9]. Perpendicular vascularization is specific for papilloma, high-grade dysplastic lesions, carcinoma in situ, and invasive carcinoma [9].

The high diagnostic yield of the classification has been confirmed by other authors [10,11]. Šifrer et al. studied 104 patients and described perpendicular vascularization in only 9.3% of benign lesions [10]. Histologically verified papillomatosis and malignant lesions showed perpendicular vascularization in 96.2% of subjects [10]. Table 2 overviews this classification.

- **Classification according to Puxxedu et al. (2016) [12]**

This classification system was designed exclusively for enhanced contact endoscopy [12]. This technology combines enhanced endoscopy imaging (such as NBI or IMAGE 1S) and a special magnifying endoscope with a magnification up to 150x. Magnification of the observed tissue allows precise description of the changes in vascular microarchitecture. This technology is suitable only for use under general anesthesia due to the lack of flexible magnifying endoscopes.

**Figure 4:**
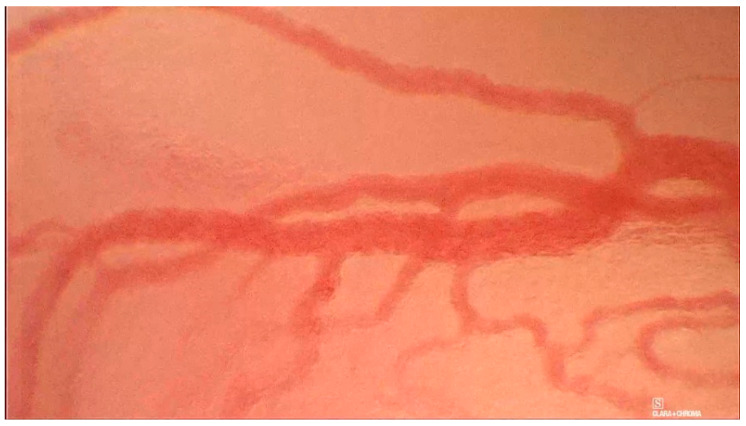
Histologically verified polyp of the left vocal cord. Puxxedu classification type I.

**Figure 5:**
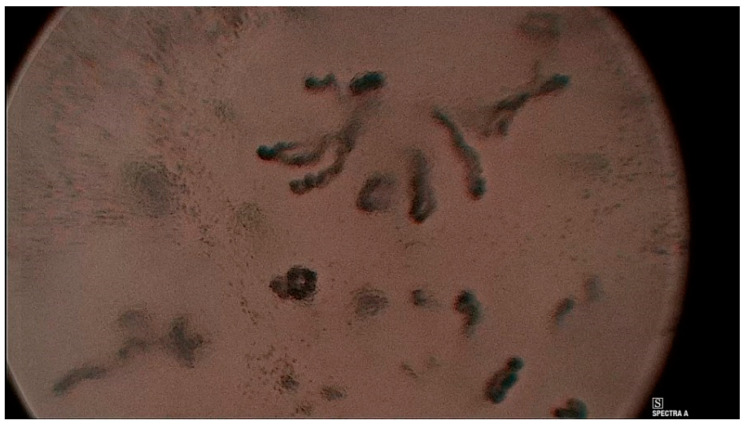
Histologically verified supraglottic squamous cell carcinoma. Puxxedu classification type IV.

The classification separates mucosal findings into types 0-IV, where 0 means normal mucosa, type I is interpreted as an inflammatory lesion, and type II is hyperplasia or papillomatosis if the capillary loop is encased by mucosal papilloma (Figure 4) [12]. Type III implies mild to moderate dysplasia [12]. Type IV should be interpreted as either high-grade dysplasia, carcinoma in situ, or invasive carcinoma (Figure 5) [12]. The results provided by Puxxedu et al. are promising and suggest that the sensitivity and specificity of the method in differentiating normal tissue vs. histological alterations is 100% [12]. The same sensitivity and specificity were achieved for differentiation of normal and inflammatory lesions vs. invasive carcinoma [12]. To differentiate between normal tissue and hyperplasia vs. dysplasia and invasive carcinoma, Puxxedu found a sensitivity and specificity of 97.6% [12]. We could not find other studies that confirm or contradict the results of this study. The overview of this classification can be seen in Table 3.

### 3.2. Classification Systems Used in Examination of Leukoplakia

Leukoplakia represents a specific diagnostic and therapeutic problem, and thus particular classification systems for describing this distinct pathology have been developed. Leukoplakia is a descriptive term used to name white patch-like lesions present on the mucosa [13]. Leukoplakia of the larynx can be mostly observed on the vocal cords. It is caused by extensive irritation of the laryngeal mucosa by alcohol, smoking, voice overuse, or laryngopharyngeal reflux [13]. The irritation causes formation of a keratin layer. Another cause of laryngeal leukoplakia is the use of inhalation corticosteroids [14]. Even though the term leukoplakia has been used for decades, it is descriptive but not clinically useful because it does not provide the risk stratification of the lesion. Histologically, the lesions can vary from hyperkeratosis to invasive cancer [15]. Therefore, early identification of the character of the lesion is crucial for a good prognosis and outcome of the treatment.

The pre-histological diagnosis of leukoplakia is difficult. Even though as much as 50% of the samples return as non-dysplastic lesions from the histopathology exam, a diagnosis of invasive cancer is made in 6–22% of the samples [16,17,18]. Therefore, lesion biopsy under general anesthesia remains common practice.

A few classification systems have been developed, and some of them can be used with white light endoscopy while others require enhanced imagining such as NBI. However, the proper NBI examination is difficult and sometimes impossible due to the “umbrella effect” [13]. This phenomenon causes the reflection of the light emitted from the light source. Therefore, the emitted light does not reach the IPCLs in the mucosa, which limits examination [13]. Nevertheless, vascularization around the leukoplakia can be observed and can yield important information about the observed lesion. It can be classified according to one of the available classifications. According to multiple authors, changes in the vascular architecture surrounding the primary lesion yield valuable information about the features of the lesion [13,19]. Stanikova et al. reported that perpendicular vascularization surrounding the leukoplakia was associated with malignant lesions (carcinoma in situ or invasive carcinoma). This was histologically confirmed in 84.6% of cases [19]. Leukoplakia surrounded by longitudinal type of vascularization was histologically benign (hyperkeratosis or low-grade dysplasia) in 83.8% of cases [19]. The authors also suggest that leukoplakia with favorable surrounding findings in NBI endoscopy can be followed conservatively without surgical intervention [19].

- **Clinical scoring of leukoplakia according to Young et al. (2014) [20]**

Young et al. proposed a scoring system of vocal cord leukoplakia based on their macroscopical appearance during white light endoscopy [20]. His classification stratifies leukoplakia by seven macroscopical features: color, texture, size, hyperemia, thickness, symmetry, and oedema [20]. Color, texture, size, and hyperemia significantly correlated with final histopathology and therefore were proposed as one of the possible ways to select high-risk patients. Interrater reliability of the classification was found to be from 68 to 79% [20]. Lesions with lower scores had very high probability to be less aggressive and should be managed conservatively [20]. Unfortunately, the study did not provide an optimal cut-off point that could be used to differentiate between low-risk and high-risk lesions. The overview of this classification can be seen in Table 4.

- **Clinical scoring of leukoplakia by Fang et al. (2016) [21]**

Fang et al. continued the previous research and removed one of the criteria (edema) from the Young et al. scoring system. Therefore, a six-tier system was established. Observed morphological features of the leukoplakia were useful in differentiation between malignant and benign lesions [21]. The morphological features were color, texture, size, hyperemia, thickness, and symmetry. The scoring system achieved good sensitivity (80.4%) and specificity (81.5%) with good interrater reliability [21]. Unfortunately, this study did not provide a specific cut-off that could be used to differentiate between benign and malignant lesion. Rather, the authors advised clinicians to set the cut-off point for each institution individually [21]. The overview of this classification can be seen in Table 5.

- **Laryngoscopic classification of vocal cord leukoplakia by Zhang et al. (2017) [17]**

**Figure 6:**
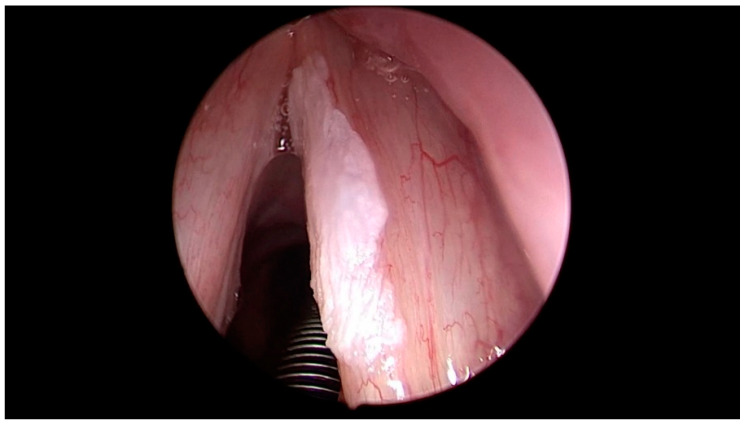
Histologically-verified parakeratosis of the right vocal cord presenting as leukoplakia. Young scoring system—3 points, Fang scoring system—3 points, Zhang type I—flat and smooth.

**Figure 7:**
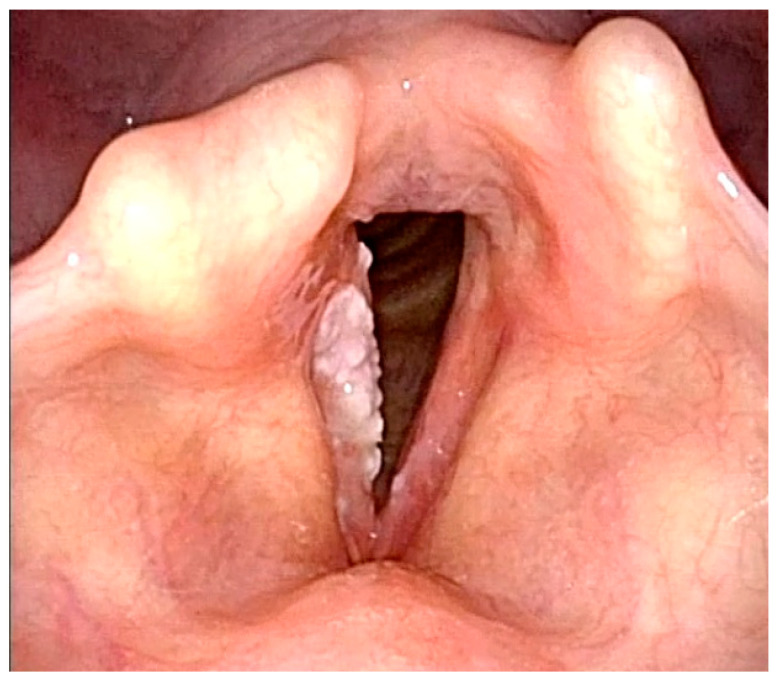
Histologically-verified squamous cell carcinoma of the right vocal cord presenting as leukoplakia. Young scoring system—5 points, Fang scoring system—5 points, Zhang type III—bulge and rough.

Zhang et al. tried to simplify classifications mentioned before by stratifying vocal cord leukoplakia into three subtypes: type I—flat and smooth; type II—bulged and smooth; and type III—bulged and rough [17]. According to the results, type I is mostly histologically interpreted as keratinization or hyperplasia without dysplastic changes (Figure 6) [17]. In type II, the dominant histology was mild to moderate dysplasia [17]. Type III presented the highest incidence of cancerous lesion (carcinoma in situ or invasive carcinoma), while incidence of non-cancerous lesions (keratosis or hyperplasia) was the lowest from all types (Figure 7) [17]. The authors further proposed conservative treatment in type I leukoplakia and surgical resection in type III leukoplakia [17]. Type II remains a grey zone, but the authors stated that leukoplakia in this stage is irreversible and may contain moderate or severe dysplasia [17]. The overview of this classification can be seen in Table 6.

A similar classification system was also proposed by Chen et al. [22]. This classification also used a three-tier classification system with similar categories: flat and smooth, elevated and smooth, and rough leukoplakia [22]. This study included 375 patients treated for vocal cord leukoplakia and confirmed that the morphology of the leukoplakia correlates significantly with the final histology examination [22].

- **Narrow-Band Imaging endoscopic classification of laryngeal leukoplakia according to Ni et al. (2019) [23]**

**Figure 8:**
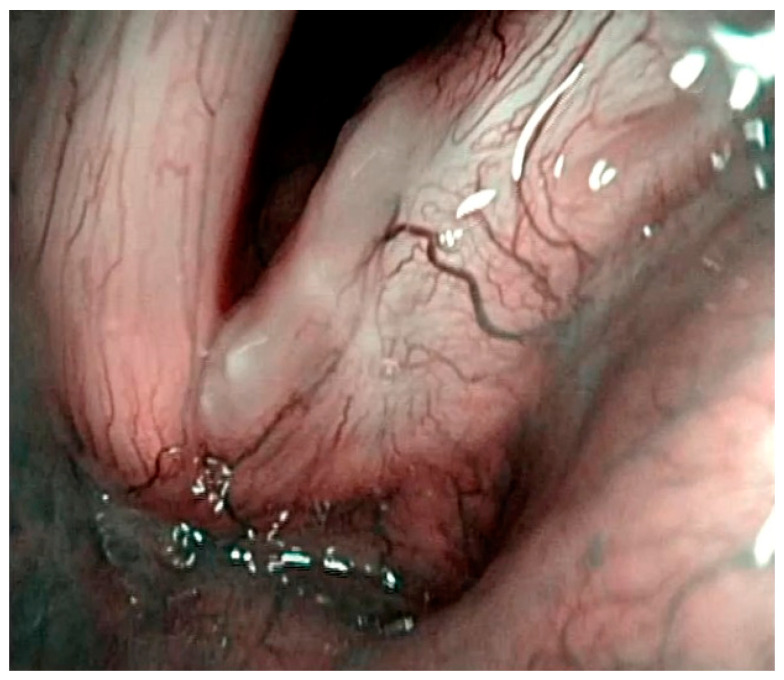
Histologically-verified low-grade dysplasia presenting as leukoplakia of the left vocal cord. Ni classification of laryngeal leukoplakia—type I.

**Figure 9:**
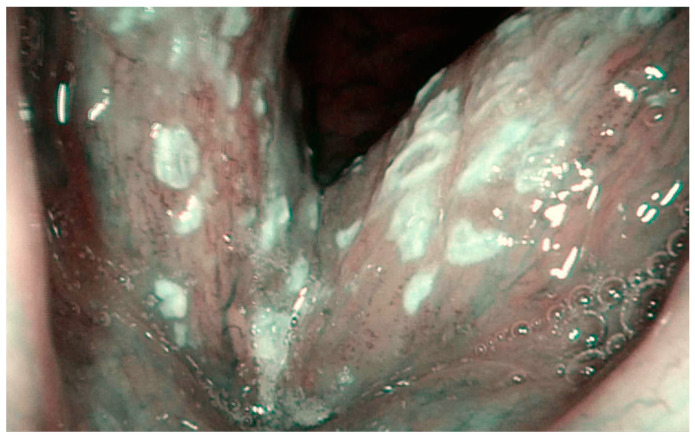
Histologically-verified squamous cell carcinoma presenting as leukoplakia of both vocal cords. IPCLs can be seen around leukoplakia. Ni classification of laryngeal leukoplakia—type III–IV.

Attempts to introduce advanced endoscopic methods used the modified Ni et al. classification. This classification stratifies leukoplakia into six types. Types 1–3 indicate benign leukoplakia (Figure 8) and types 4–6 suggest possibility of malignancy (Figure 9) [23]. The accuracy of the classification in judging the pathological nature of the leukoplakia was 90.8% [23]. The overview of this classification can be seen in Table 7.

An examination that can provide additional information about the lesion is laryngeal videostroboscopy. According to Rzepakowska et al., non-invasive leukoplakia (parakeratosis, low-grade dysplasia, etc.) tends to preserve the mucosal wave of the vocal cord [24]. On the other hand, the mucosal wave tends to diminish in the case of an invasive form of leukoplakia (high-grade dysplasia, invasive carcinoma, etc.) [24]. As stated by El-Demerdash, the overall accuracy of laryngeal videostroboscopy versus histology was 95% [25]. Those results were further confirmed by studies by other authors [25,26,27].

## 4. Discussion

Every classification system carries certain advantages and disadvantages. One of the major advantages of ELS classification is its simplicity. This two-stage system allows the examinator to classify mucosal vasculature findings as either perpendicular or longitudinal. Mehlum et al. found low interrater variability and suitability of the classification for inexperienced examinators [28]. A major disadvantage of this system is that it does not try to specify what the lesion is histologically according to endoscopy findings. The question is if specification of the histology of the lesion pre-operatively is required.

Ni classification provides ample information about the nature of the lesion. It also tries to state its histological character according to endoscopy findings. Unfortunately, the Ni classification has a few disadvantages. The major problem is the blurry cut-off line between malignant and benign lesions. According to the classification, Ni IV IPCLs have the appearance of small and dark brown spots, and should be interpreted as benign lesions [2]. Unfortunately, this appearance of IPCLs would be interpreted as perpendicular and therefore suspect according to ELS classification [9]. Therefore, a study determining whether Ni IV should be interpreted as a benign or suspect lesion is required. Another problem that affects multiple classification systems is the use of the old classification of laryngeal dysplasia. The terms “mild”, “moderate”, and “severe dysplasia” should no longer be used according to the new World Health Organization (WHO) revision of laryngeal dysplasia terms [29]. These terms should be replaced and reclassified to low-grade dysplasia and high-grade dysplasia according to the WHO [29].

The Puxxedu classification for ECE yields interesting data—it provides histological specifications of the examined lesion, and very high sensitivity and specificity are stated in the original work. Unfortunately, the sensitivity and specificity are calculated in a sub-optimal way in the original paper. Puxxedu stated his sensitivity and specificity rates according to healthy tissue vs. malignant tumors or inflammation changes vs malignant tumors [12]. These changes are usually very well pronounced and easy to differentiate even without ECE; therefore, the results may be biased. A study that compares sensitivity and specificity calculated according to the Puxxedu classification vs. final histology examination is therefore required. Also, the Puxxedu classification still uses the old classification of laryngeal dysplasia and should be modified to fit the new WHO recommendations.

Moreover, the situation is even more difficult if the patient has undergone radiotherapy. The vasculature is influenced by radiation and it is difficult to interpret vascular character properly. This makes differentiating between recurrence of the malignant tumors and post-radiation changes very difficult. The experience of the examiner comes into play more significantly. On the other hand, according to Zabrodsky et al., NBI is a good tool for follow-up of patients after radiotherapy for laryngeal and hypopharyngeal cancer with sensitivity of 92%, specificity of 76%, and overall accuracy of 88% [30].

Management of vocal cord leukoplakia remains a challenging topic in modern otolaryngology. Biopsy under general anesthesia and histological verification of the leukoplakia remains a common practice. Fortunately, clinicians have started to stratify the risk of malignancy of the leukoplakia using various classification systems as mentioned above to properly assess the risk of malignancy. A management algorithm combining the morphology of the leukoplakia, laryngeal videostroboscopy, and assessment of IPCLs around the lesion should be used to assess the risk of malignancy. If it remains low, then conservative treatment is suggested by some authors [19,20,22,31,32]. However, when managing the leukoplakia conservatively, clinicians should be very cautious and in case of any doubt examination under general anesthesia with histology examination of the leukoplakia should be performed.

Isenberg et al. provided a systematic review of 2188 biopsies of leukoplakia and showed that mild to moderate dysplasia was found in 33.5% of cases, and high-grade dysplasia or carcinoma in situ was found in 15.2% of cases [16]. According to Weller et al., laryngeal dysplasia carries a significant risk of malignant transformation [33]. The risk triples with increasing severity of dysplasia [33]. Therefore, clinicians should be aware of the possibility of malignant transformation of the vocal cord leukoplakia and patients should be tightly observed. Early discharge of patients with vocal cord leukoplakia should not be a common practice.

Unfortunately, we are still far from the concept of optical biopsy and pre-histology diagnosis. None of the advanced endoscopy methods can overcome histological verification of the lesion. The important point is that examinators should not assess laryngeal lesions solely according to their vasculature changes, appearance, or preservation of the mucosa wave. All available examinations should be performed to gather as much information as possible. Only complex and detailed examination allows the highest accuracy and diagnostic yield.

The future in differential diagnosis of laryngeal lesions is probably artificial intelligence (AI) and machine learning. These systems will probably be able to eliminate the problems with the subjective evaluation of the mentioned endoscopic classifications. Żurek et al. analyzed 11 studies that used AI in the early diagnosis of laryngeal lesions. Although various AI models were used, the overall accuracy was very high—from 80.6% to 99.7% [34]. The pooled sensitivity and specificity for differentiation between benign and malignant lesions were also very high: 91% and 94%, respectively [34].

## 5. Conclusions

ENT endoscopy remains a rapidly evolving and dynamic field of medicine, but the concept of optical biopsy and pre-histology diagnosis remains a challenging problem. The available classification systems provide very good sensitivity and specificity. However, the non-coherence of the classification systems remains an issue, and therefore a unified classification system is needed. Further research is needed to determine whether the Ni IV should be interpreted as a benign or a suspicious lesion. Also, research on the field of leukoplakia risk assessment is required. Artificial intelligence will probably be a valuable assistant in laryngeal examination in the future.

## Figures and Tables

**Table 1:** Narrow-band imaging endoscopic classification of the laryngeal lesions according to Ni et al. (2011) [2].

Endoscopic Pattern	Morphology of Vessels	IPCLs
Type I	Small, oblique, and arborescent	Not visible
Type II	Enlarged, oblique, and arborescent	Not visible
Type III	Obscured or seen indistinctly by white mucosa	Not visible
Type IV	Oblique and arborescent vessels not visible	Small and dark brown spots
Type Va	Oblique and arborescent vessels not visible	Dilated, solid, or hollow, with a brownish, speckled pattern, and various shapes
Type Vb	Oblique and arborescent vessels not visible	Tortuous, irregular, with a snake, earthworm, tadpole, or branch-like shapes
Type Vc	Oblique and arborescent vessels not visible	Tortuous or brownish speckles with irregular distribution

Abbreviation: IPCLs—intrapapillary capillary loops.

**Table 2:** Classification according to the European Laryngological Society by Arens et al. (2016) [9].

Endoscopic Pattern	Morphology of Vessels	
Longitudinal vascular changes	Ectasia	Dilated vessels
	Meander	Meandering, tortuous vessels
	Varicose	Advanced meandering and dilated vessels
	Convolute	Organized coil/tangle of vessels
	Number of vessels	Increased vessels number
	Branches of vessels	Increased branches of vessels
	Change of direction	Abrupt change of vessels direction
Perpendicular vascular changes	Enlarged vessel loops	Abnormal IPCLs with wide-angled turning points
	Dot-like vessel loops	Abnormal IPCLs with narrow-angled turning points
	A Worm-like vessels	Abnormal vessels with spiral morphology and bizarre course

Abbreviation: IPCLs—intraepithelial capillary loops.

**Table 3:** Classification according to Puxxedu et al. (2016) [12].

Vascular Pattern	Diagnosis	Description
Type 0	Normal mucosa	Thin-end regular subepithelial vessels connecting with a thicker and deeper arborescent vascular network running parallel to the epithelium.
Type I	Inflammation	The subepithelial vessels are increased in number and size with irregular and sometimes crossing directions.
Type II	Hyperplasia	Intra-CLs are visible running toward the surface when the hyperplasia is at the initial stage. In this phase, CLs are generally still very thin and short, arising from the underlying inflammatory vasculature with a scattered distribution. In the case of mature hyperplasia, the deeper inflammatory vascular network is not visible, and only the elongated CLs can be easily seen. In the case of vegetating keratosis, the deeper inflammatory vascular network is often not visible, and the elongated CLs are difficult to see. A particular type of “bobby-pin” can be seen in laryngeal papillomatosis. The typical papilla encases the “bobby-pin” inside the papilloma.
Type III	Mild–moderate dysplasia	Vascular changes become progressively more consistent with elongated small vessels in the typical “bobby-pin” shape, but some arborescence appears at the end of the CLs.
Type IV	High-grade dysplasia/carcinoma in situ/invasive carcinoma	The vascularity of the chorion is more evident and CLs appear significantly dilated with various shapes and a wide range of vascular architectural changes such as corkscrews or tree-like patterns.

Abbreviation: CLs—capillary loops.

**Table 4:** Clinical scoring of leukoplakia according to Young et al. (2014) [20].

Factors	Categories	Score	Definitions of the Vocal Cord Leukoplakia
Color	Homogenous	0	The color is distributed evenly.
	Non-homogeneous	1	The color is not distributed evenly.
Texture	Regular	0	The surface is smooth and flat.
	Irregular	1	The surface showed granular appearance.
Size	Small	0	The sum of all vocal cord leukoplakia is less than half a length of one true vocal cord.
	Large	1	The sum of all vocal cord leukoplakia exceeds half a length of one true vocal cord.
Hyperemia	Absence	0	The vocal cord leukoplakia is without peripheral erythema or increased vascularity.
	Presence	1	The vocal cord leukoplakia is associated with peripheral erythema or increased vascularity.
Thickness	Thin	0	The lesion is thin and blood vessels beneath the lesion are visible.
	Thick	1	The lesion is thick and blood vessels beneath the lesion are invisible.
Symmetry	Symmetric	0	Lesions are distributed at similar sites of bilateral vocal cords.
	Asymmetric	1	Lesions are located at one or unopposed sites.
Edema	Exist	0	Existence of vocal edema.
	Absence	1	Absence of vocal edema.

**Table 5:** Clinical scoring of leukoplakia according to Fang et al. (2016) [21].

Factors		Score	Definitions
Color	Homogenous	0	The color of vocal cord leukoplakia is distributed evenly.
	Heterogeneous	1	The color of vocal cord leukoplakia is not distributed evenly.
Texture	Regular	0	The surface of vocal cord leukoplakia is smooth and flat.
	Irregular	1	The surface of vocal cord leukoplakia showed granular appearance.
Size	Small	0	The sum of all vocal cord leukoplakia is less than half length of one true vocal cord.
	Large	1	The sum of all vocal cord leukoplakia exceeds the half length of one true vocal cord.
Hyperemia	Absence	0	The vocal cord leukoplakia is without peripheral erythema or increased vascularity.
	Presence	1	The vocal cord leukoplakia is associated with peripheral erythema or increased vascularity.
Thickness	Thin	0	The lesion is thin and blood vessels beneath the lesion are visible.
	Thick	1	The lesion is thick and blood vessels beneath the lesion are invisible.
Symmetry	Symmetric	0	Lesions are distributed at similar sites of the bilateral cords.
	Asymmetric	1	Lesions are located at one or unopposed sites.

**Table 6:** Laryngoscopic classification of vocal cord leukoplakia by Zhang et al. (2017) [17].

Type of Lesion		Description
Type I	flat and smooth	Localized white plaque lesion having a uniform thin smooth homogeneous surface or white patch is raised slightly, but the edge of the white patch is continuous with the surrounding mucosa.
Type II	bulge and smooth	White plaque lesion is homogeneous and significantly bulged with a constant texture throughout. It is higher than the mucosa around the plaque. The edge of the white patch is discontinuous with the surrounding mucosa.
Type III	bulge and rough	Grayish-white, nodular, verrucous, granular, non-homogeneous, and (or) exophytic lesions with irregular blunt or sharp projections. They have an irregular surface associated with erosion or ulceration that is higher than the mucosa around the plaque.

**Table 7:** Narrow-band imaging endoscopic classification of laryngeal leukoplakia according to Ni et al. (2019) [23].

Type	Interpretation	Description
Type I	Benign leukoplakia	There are no IPCLs but white plaque can be observed on the vocal cord with obliquely running vessels and branching vessels indistinctly present under the white plaque.
Type II	Benign leukoplakia	There are white patches on the vocal cord but neither IPCLs nor obliquely running vessels or branching vessels can be found.
Type III	Benign leukoplakia	IPCLs can be seen at the surface of the vocal cord mucosa where the epithelium is not covered by the leukoplakia, thus showing small brown spots with a relatively regular arrangement without clear boundaries. No obliquely running vessels or branching vessel were seen.
Type IV	Malignant leukoplakia	IPCLs can be observed on the vocal cord, showing large brown spots embedded at the surface of white plaque.
Type V	Malignant leukoplakia	IPCLs on the vocal cord can be seen with large brown spots that appear at the surface of the vocal cord mucosa outside the leukoplakia with obvious boundaries.
Type VI	Malignant leukoplakia	IPCLs are visible at the surface of the vocal cord and are characterized by large brown spots or twisted earthworm-like vessels distributed at the surface of the leukoplakia as well as on the surface of the vocal cord epithelium outside the leukoplakia.

Abbreviation: IPCLs—intrapapillary capillary loops.

## Data Availability

Data sharing not applicable.
